# Supplementary material for: Pervasive male-biased expression throughout the germline-specific regions of the sea lamprey genome supports key roles in sex differentiation and spermatogenesis
Source: Commun Biol. 2022 May 10;5:434. doi: 10.1038/s42003-022-03375-z (PMC9090840; doi:10.1038/s42003-022-03375-z)
Supplement: Supplementary file 2 — Supplementary Information [file 42003_2022_3375_MOESM2_ESM.pdf]

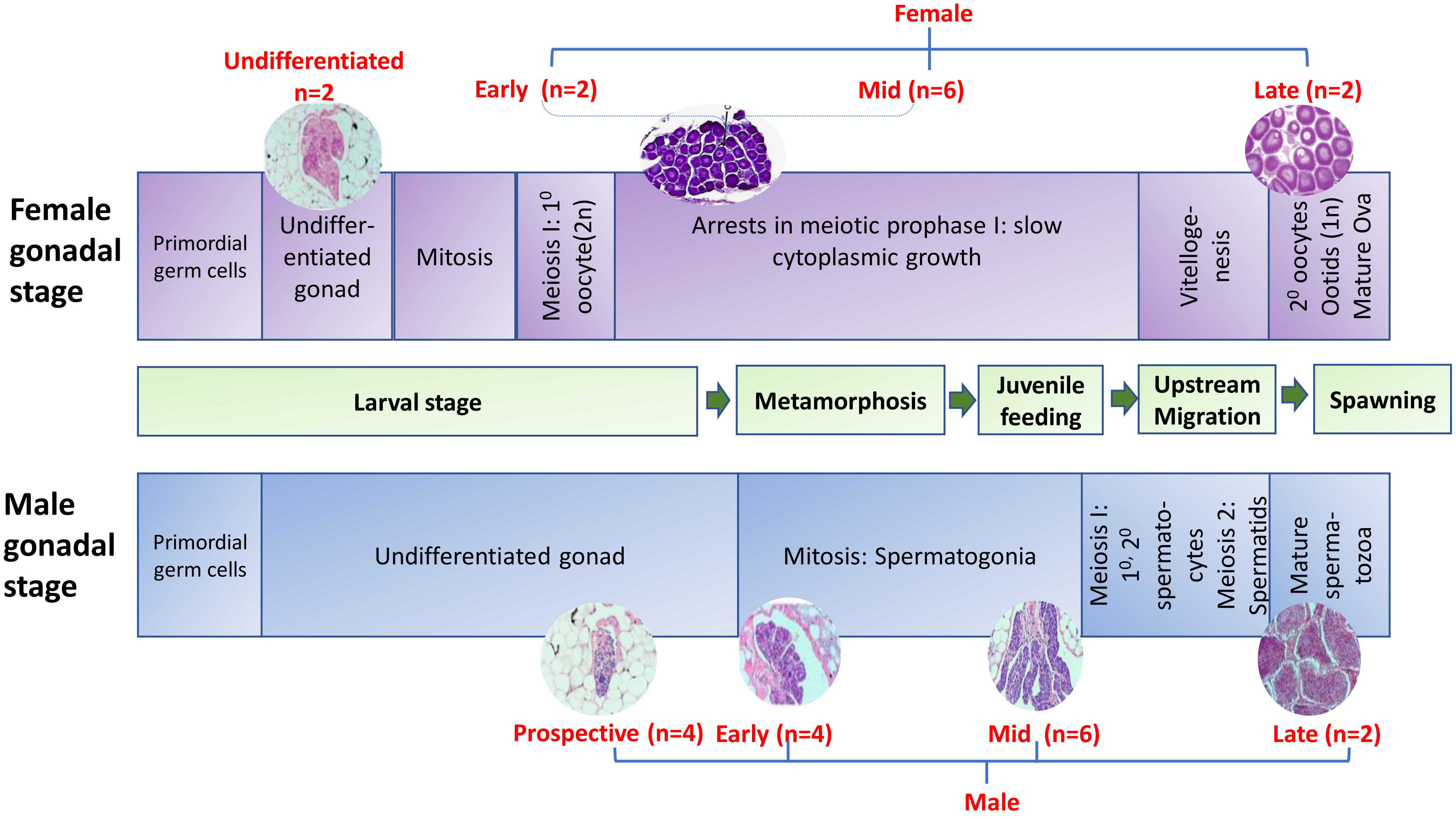

Supplementary Fig. 1: Schematic presentation of sea lamprey gonadal and life-history stages examined in this study; sample collection details are provided in Supplementary Table 1. The figure is adapted from Docker et al. (2019), with images from Khan (2017).

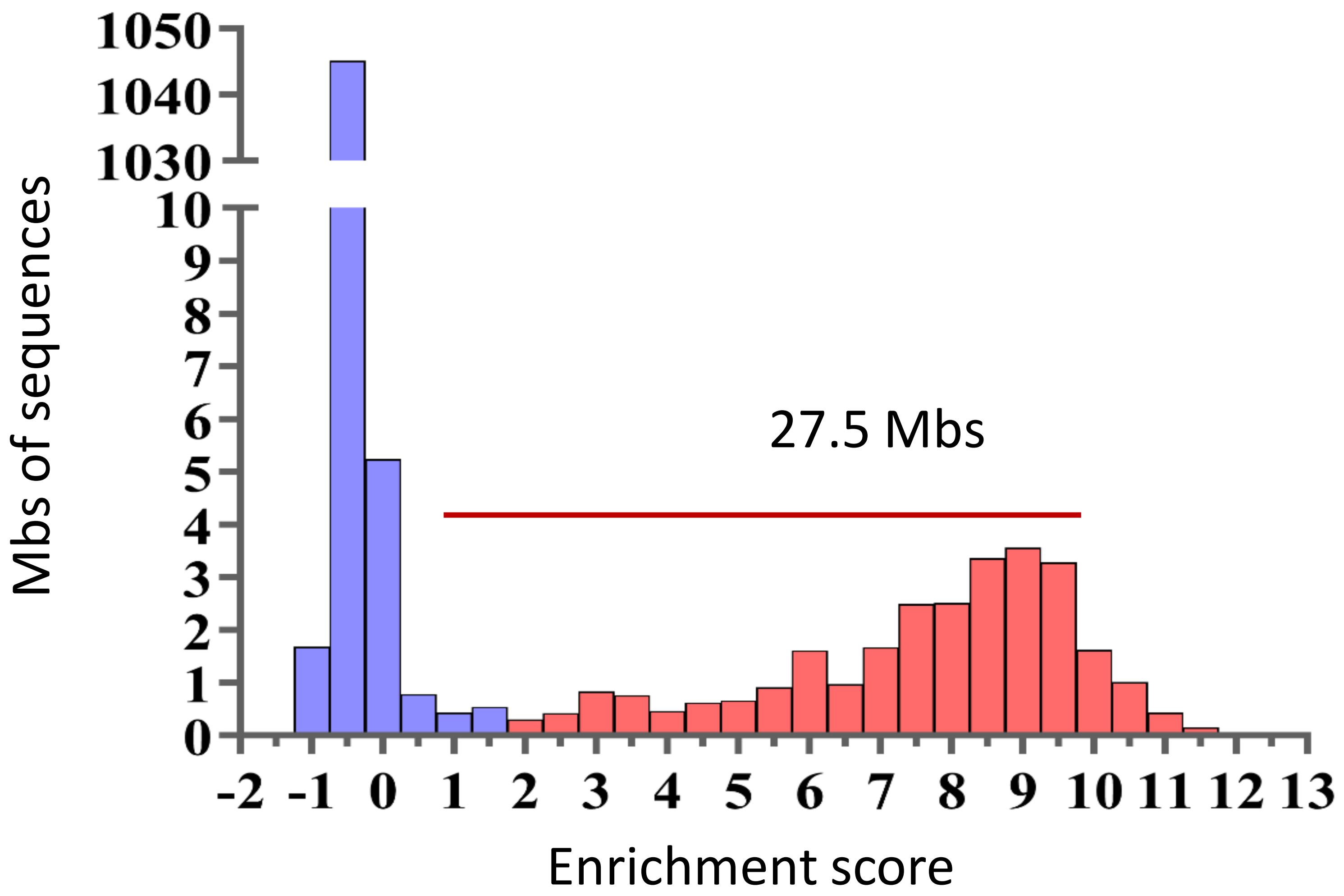

Supplementary Fig 2: Distribution of the log2(standardized sperm/blood read coverage): Bars highlighted in red correspond to regions (in Mbps) with 2-fold or greater read coverage in sperm compared to blood (assigned to GSR); blue bars to regions putatively in the somatic genome (coverage <2).

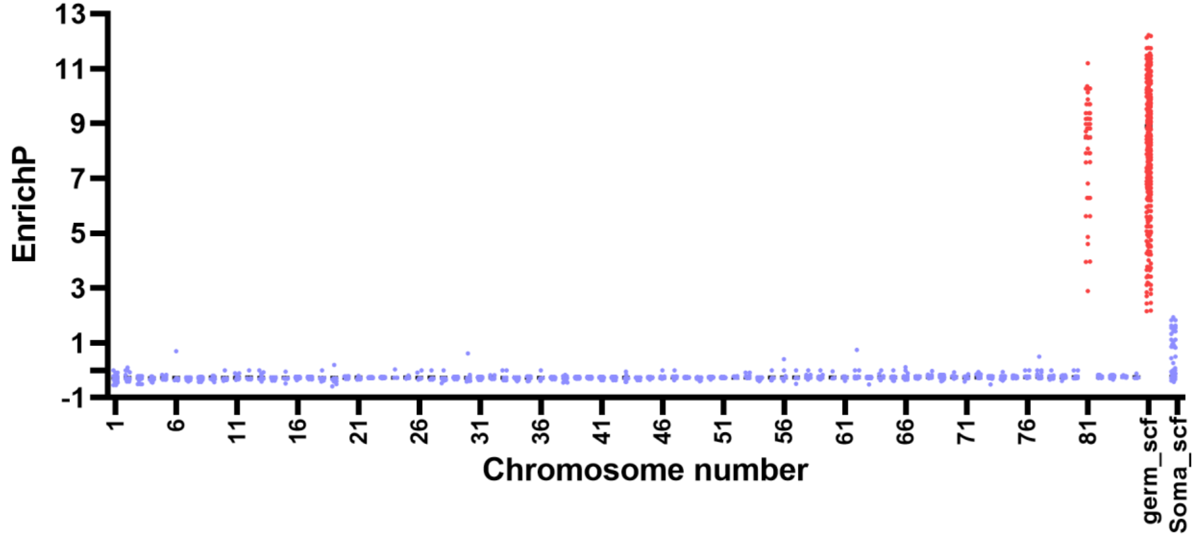

Supplementary Fig. 3: Distribution of enrichment score across genome. X-axis represents the chromosome locations and Y axis represents the the enrichment score in each chromosomal region. Chromosome 81 and germ_scf, highlighted in red, represent the region where the enrichment score was 2 or higher. If the enrichment score is less than 2, it is highlighted in purple.

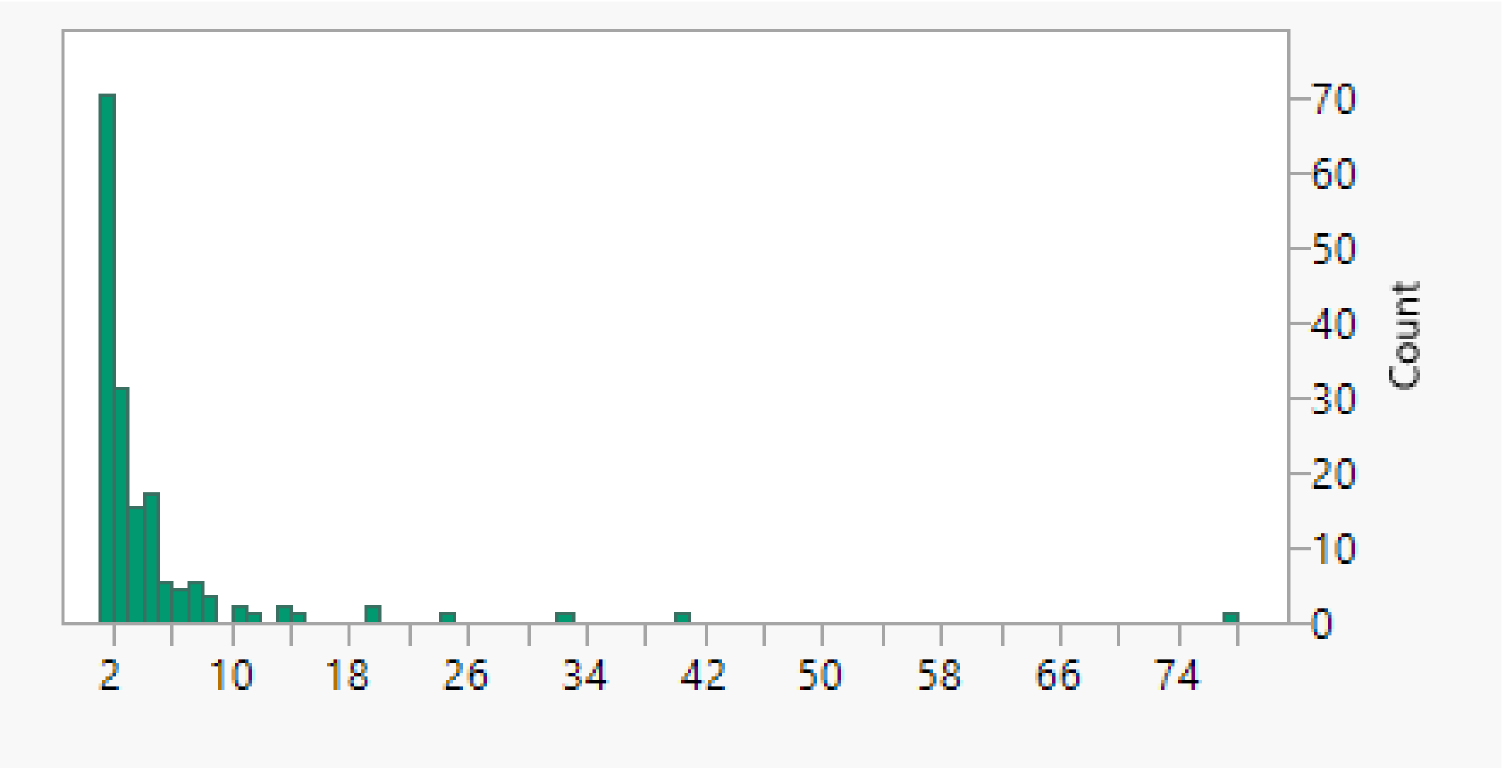

Supplementary Fig. 4: Histogram of the number of copies of GSGs in the GSR. X-axis is the frequency of a given GSGs in GSR while the Y-axis is the number of GSGs with that frequency.

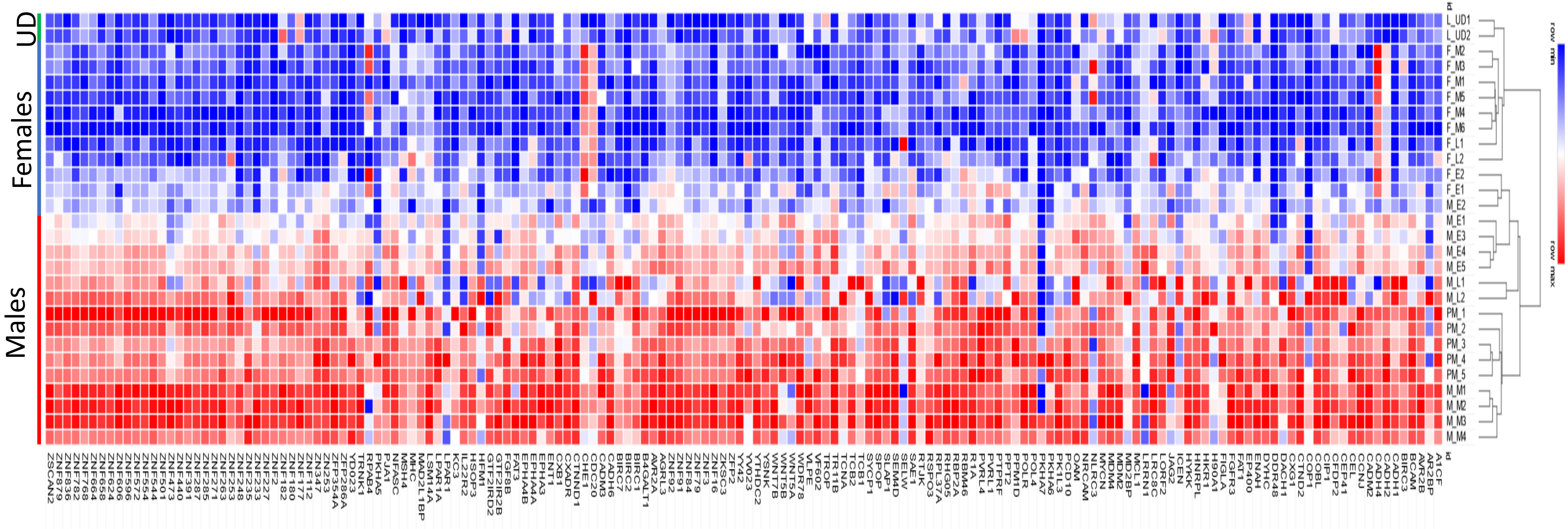

Supplementary Fig. 5: Heatmap for all GSGs in 28 gonad samples showing differences in expression among stages. UD stands for undifferentiated larvae

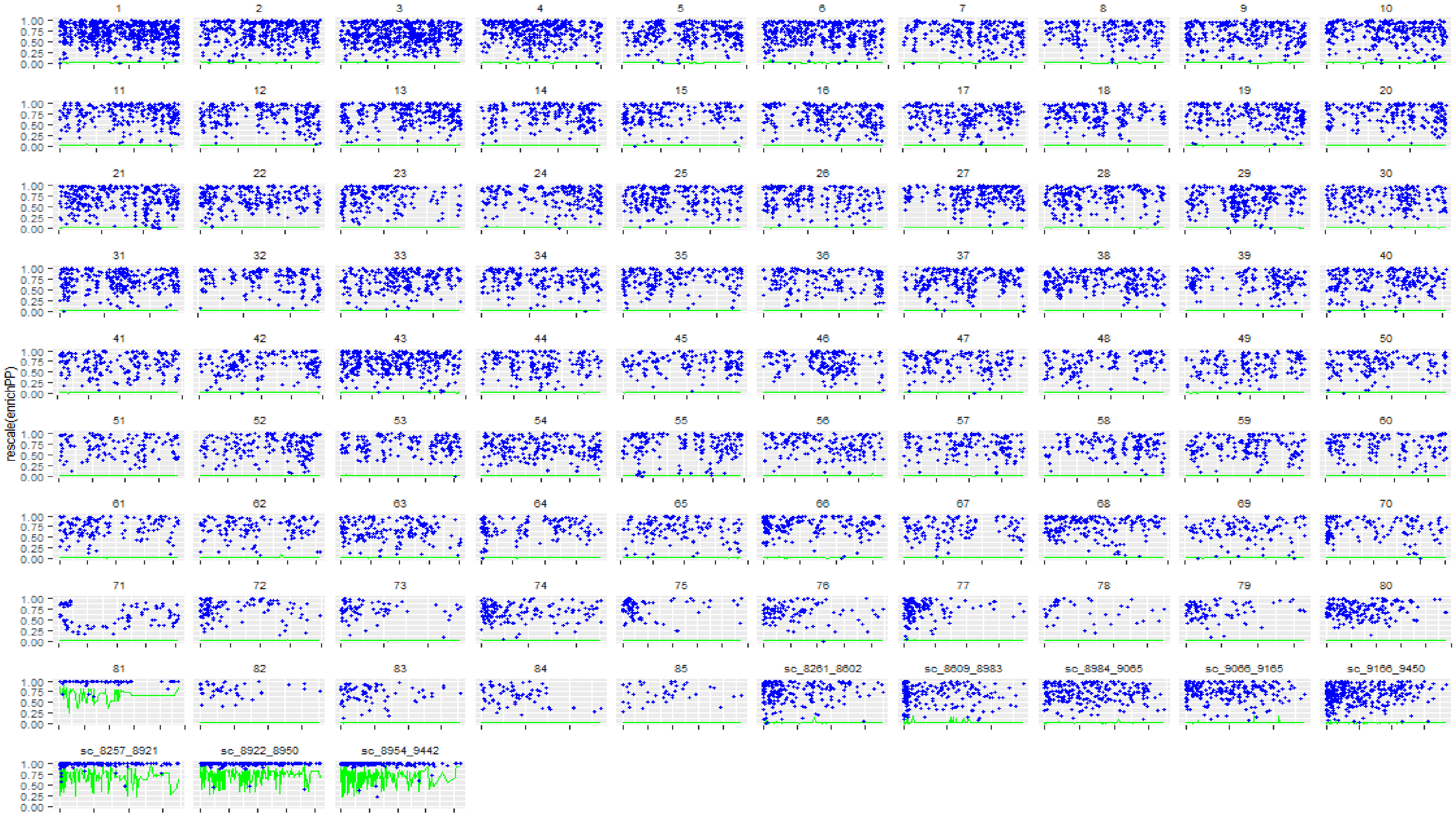

Supplementary Fig. 6: Proportion of male-biased genes in different chromosomal regions of sea lamprey genome. Y axis represents the proportion in male vs female. The proportion scale 0-1 refers to the scale of male-biasness compared to females. X axis represents the genes present in those regions. The green line represents the enrichment score of each gene across genome.

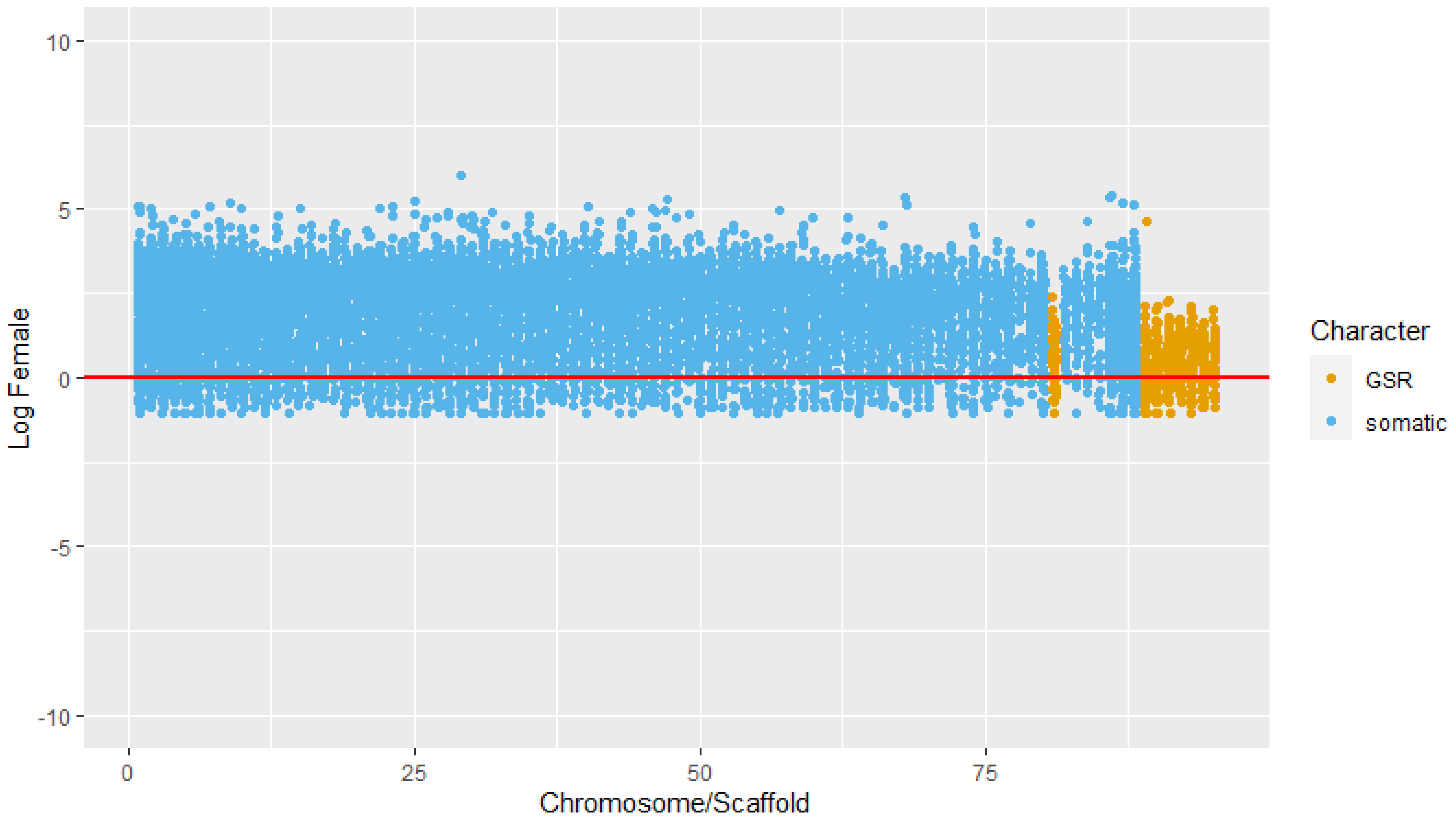

Supplementary Fig. 7: Scatterplot showing the $\log_{10}$(female) normalized gene expression across all chromosomes and concatenated scaffolds in the VGP assembly of the sea lamprey genome; genes putatively located in the GSR are colored orange, while those in the somatic genome are blue.

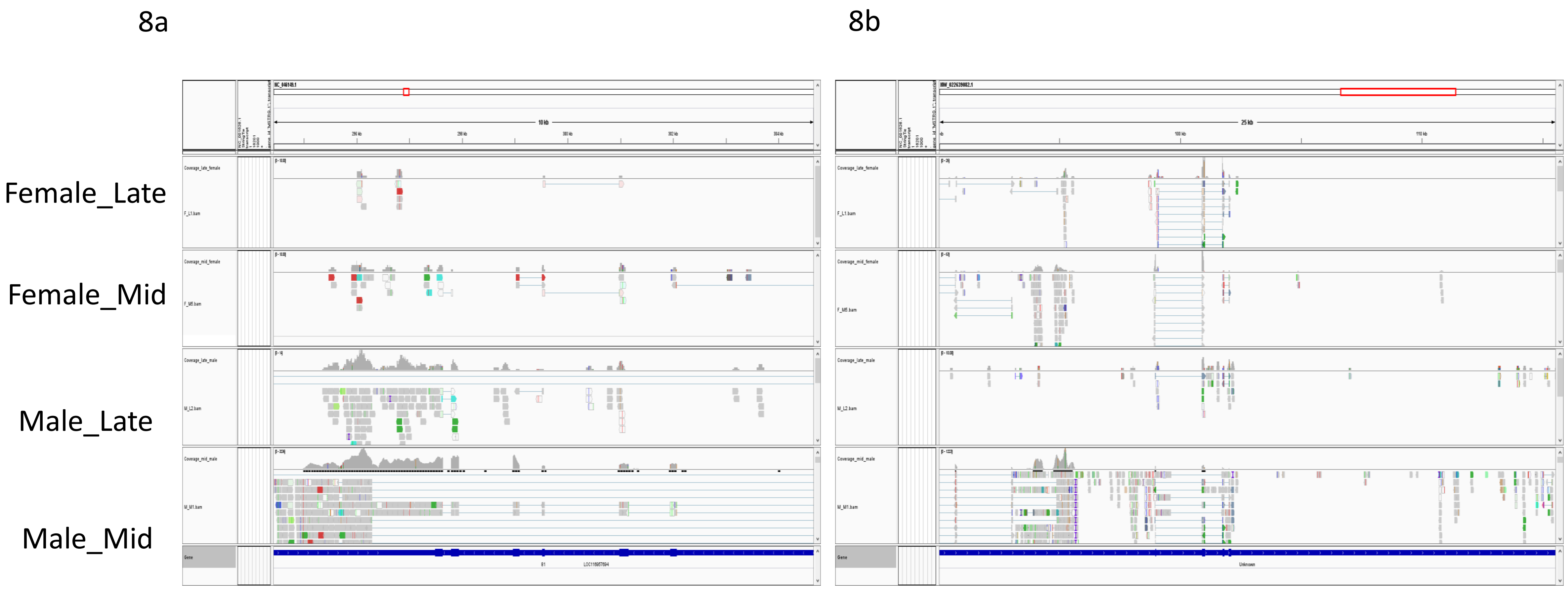

Supplementary Fig. 8: Germline-specific genes and their expression in late and mid female and males present in germline-specific region in chromosome 81 and in unplaced scaffolds in the genome viewed in IGV.
a) gene LOC116957694 b) gene LOC116937865

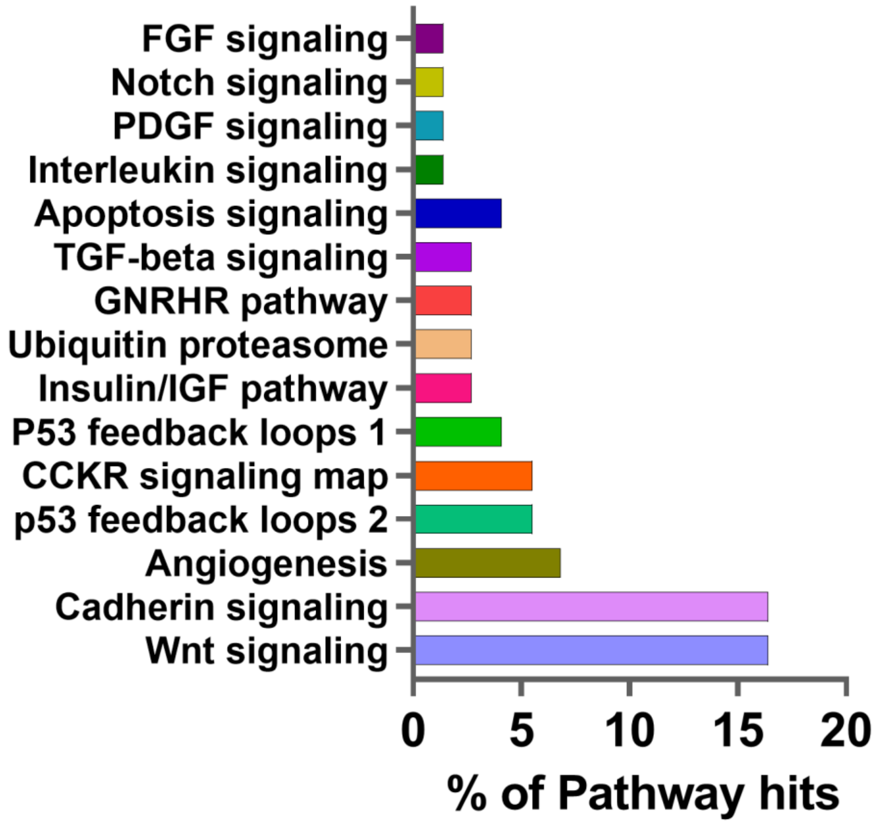

Supplementary Fig. 9: A histogram showing the pathways in which GSGs are involved along with the percentage of pathway hits. X-axis represents the percentage (%) of pathway hits and Y-axis represents different pathways involved

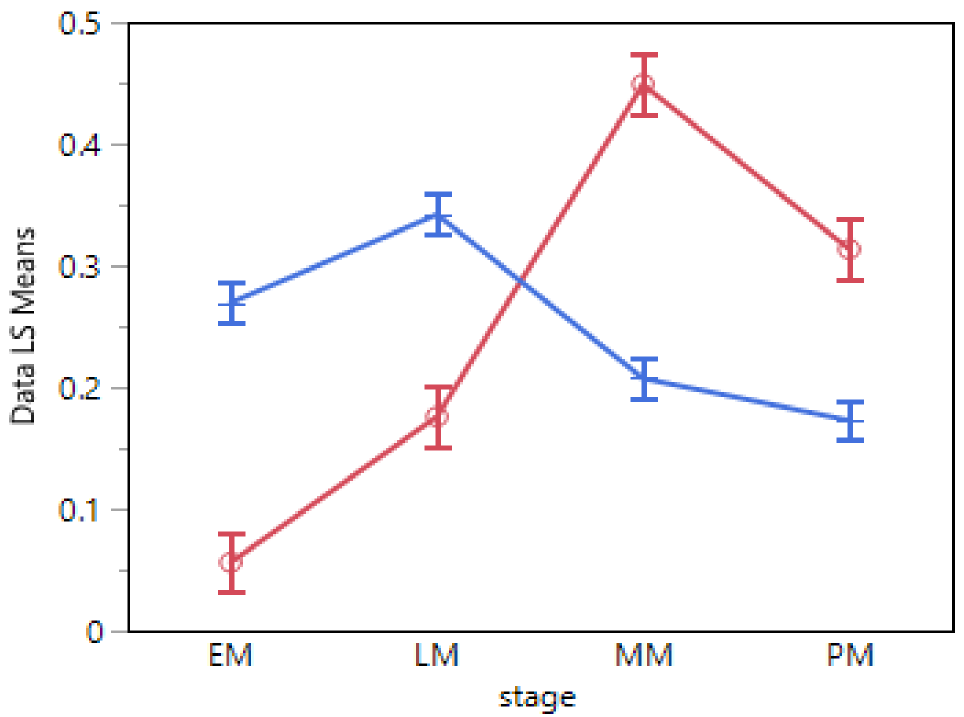

Supplementary Fig. 10: Least squares (LS) means and standard error (SE) of the mean gene expression by male gonadal stage and genome. LS means and SE were estimated from a repeated measures mixed model in which gene nested in genome was a random effect and stage was a repeated measure. Stages of male gonad development are: EM - early males, LM - late males, MM - mid males and PM - prospective males. The red and blue lines represent the LS mean estimate of gene expression by stage in the GSR and somatic genomes respectively.

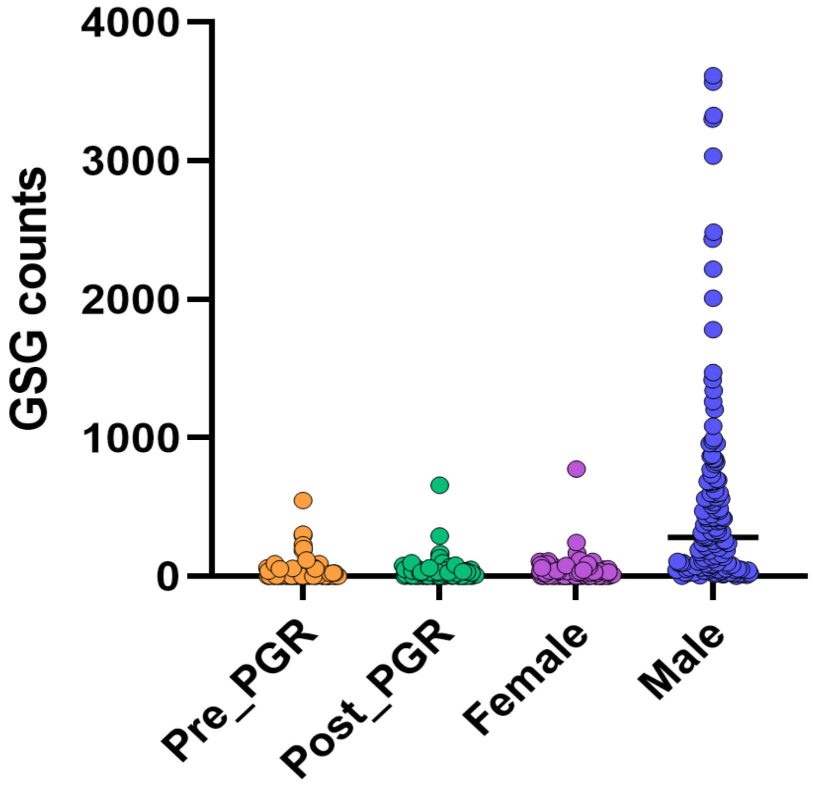

Supplementary Fig. 11: Expression of GSGs present in pre-PGR and post-PGR embryo and their comparison across stage and sexes. Y axis presents the number of GSG counts and the X axis presents pre- and post-PGR stages, including adult male and female sea lamprey. Pre-PGRs stages are 1, 2, 2.5 dpf embryos and post-PGR are 3, 4, 5 dpf embryos. Female and male GSGs counts are from this study as described in the main text.

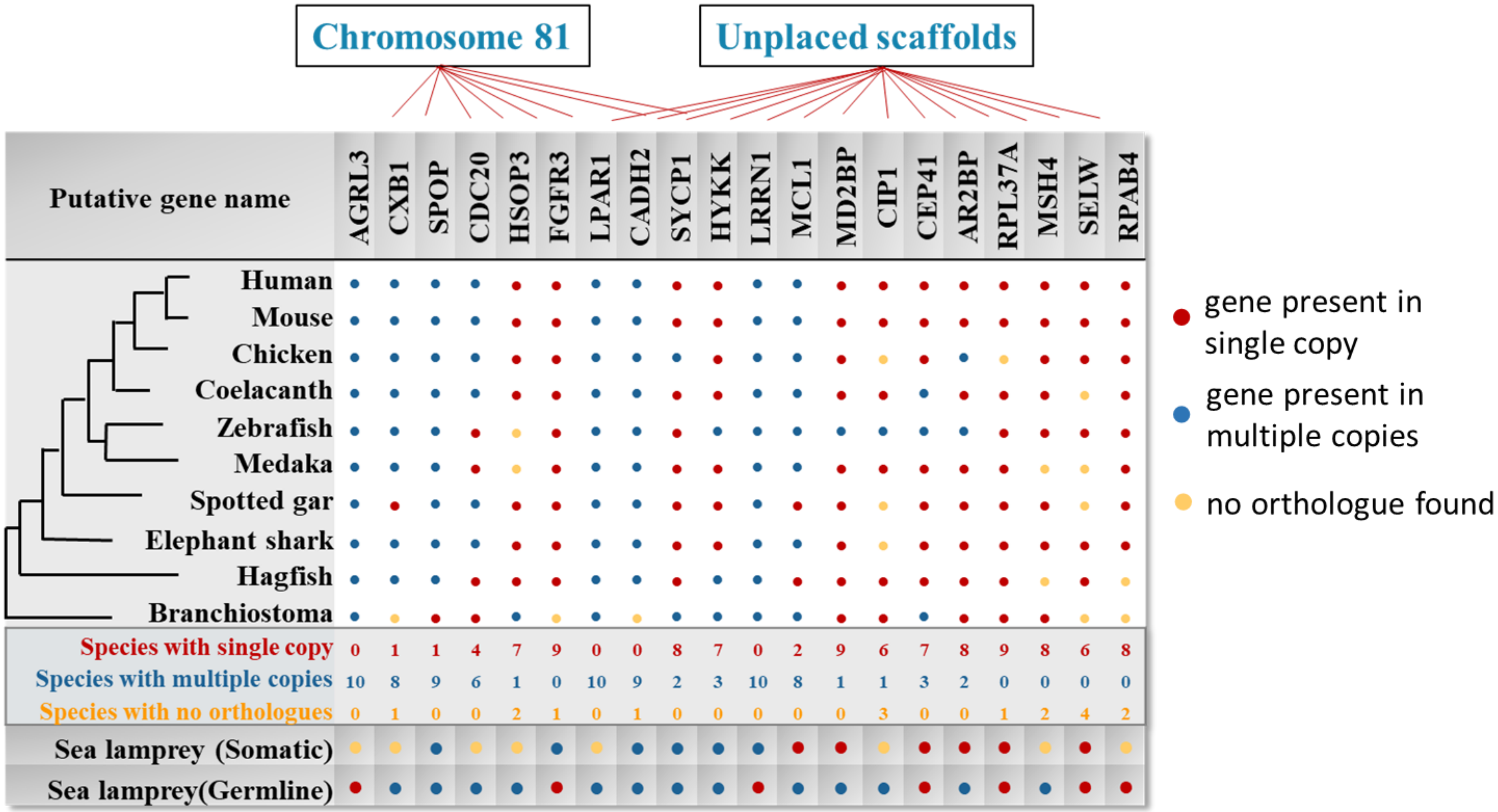

Supplementary Fig 12. Evolutionary conservation of copy number of GSGs in 11 species as inferred using OrthoFinder (see text for details); putative gene names are given across the top, the number of copies and location of putative homologs of these genes in the sea lamprey germline and somatic genomes is given below.

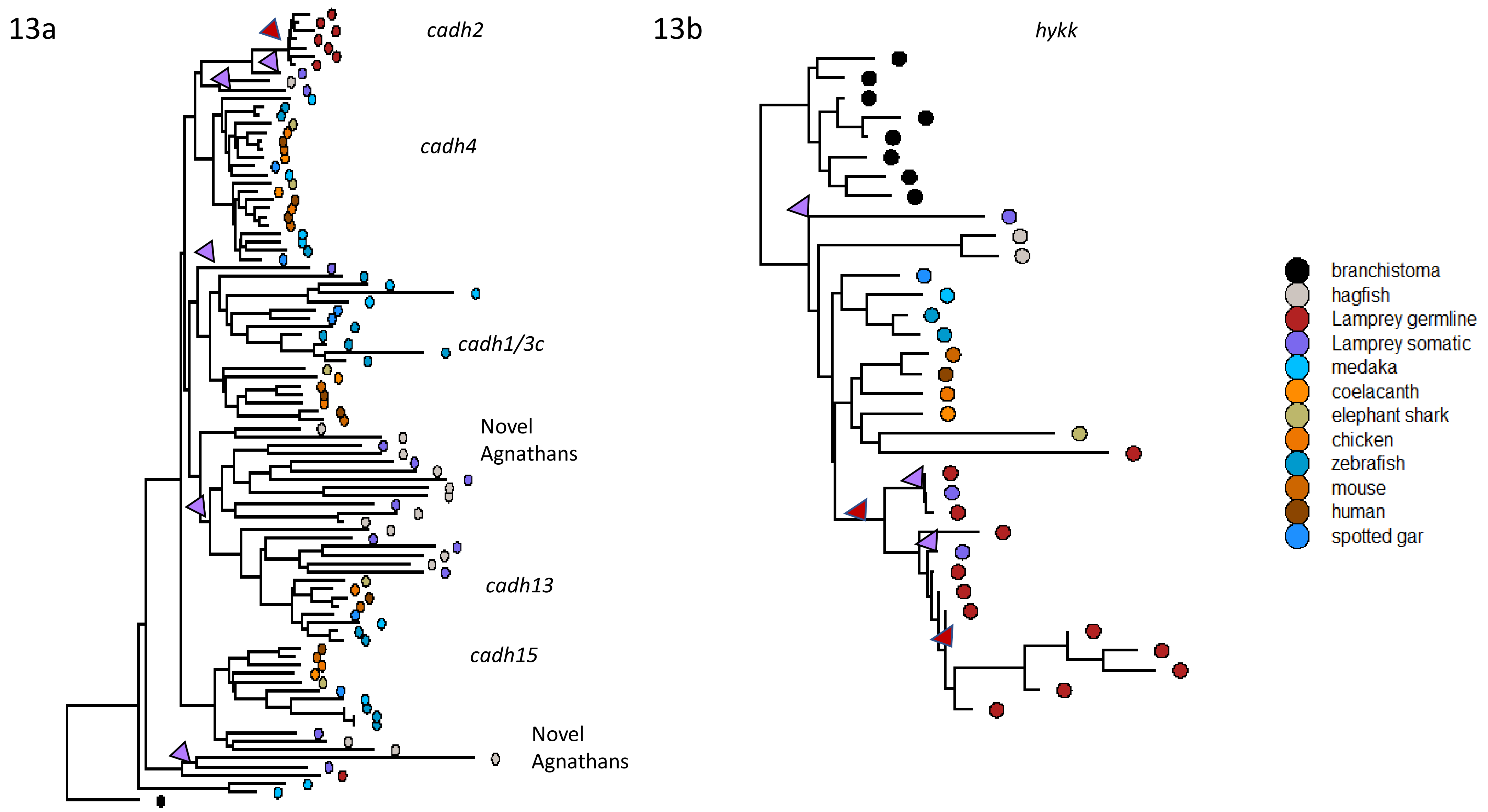

13a
cadh2
cadh4
cadh1/3c
Novel Agnathans
cadh13
cadh15
Novel Agnathans
13b
hykk
branchistoma
hagfish
Lamprey germline
Lamprey somatic
medaka
coelacanth
elephant shark
chicken
zebrafish
mouse
human
spotted gar

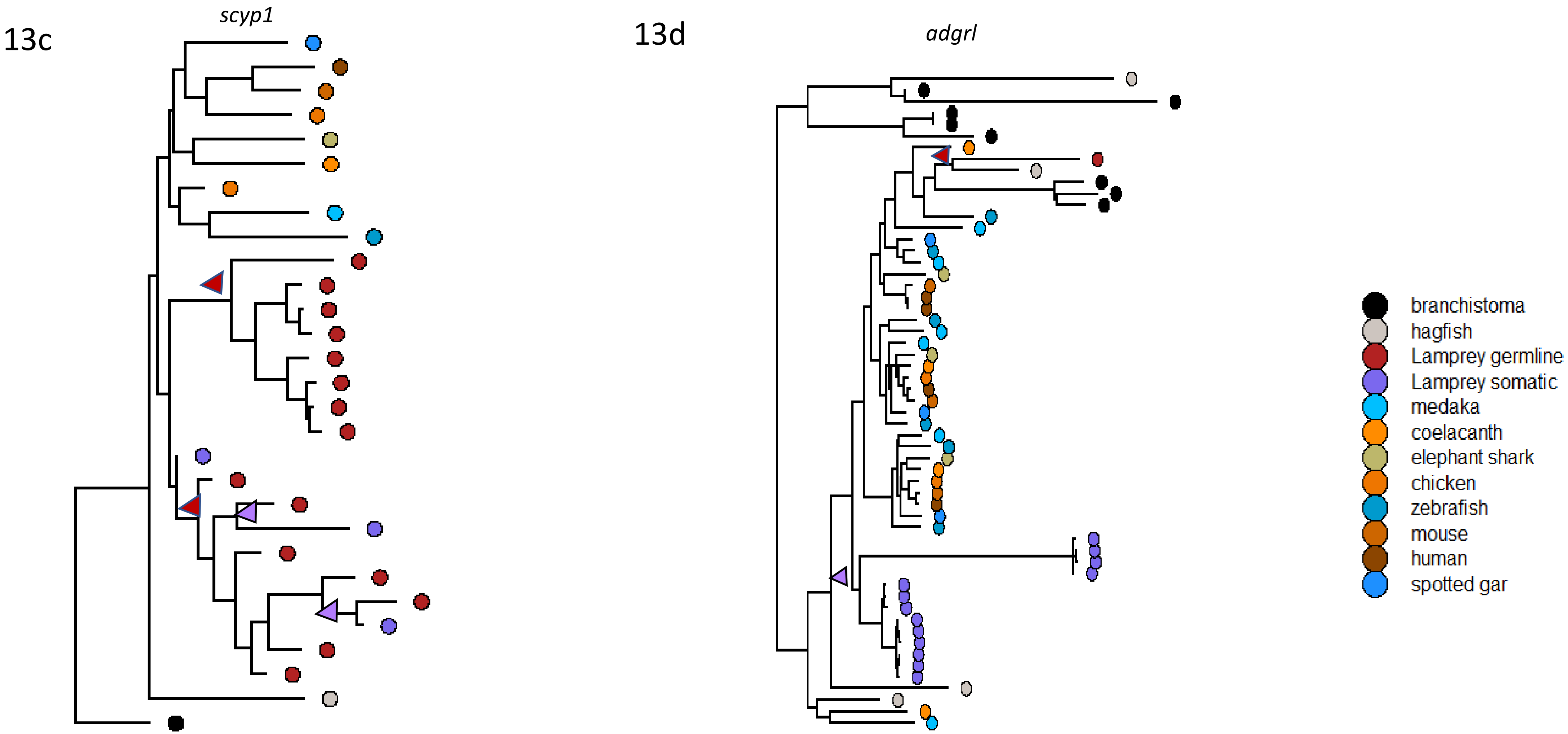

Supplementary Fig. 13: Phylogenetic tree for the gene a) *cadh*, b) *hykk*, c) *sycp1*, and d) *adgrl*. Purple triangles indicate inferred clades containing somatic paralog(s) of a gene, while red triangles indicate clades with an inferred germline paralog.

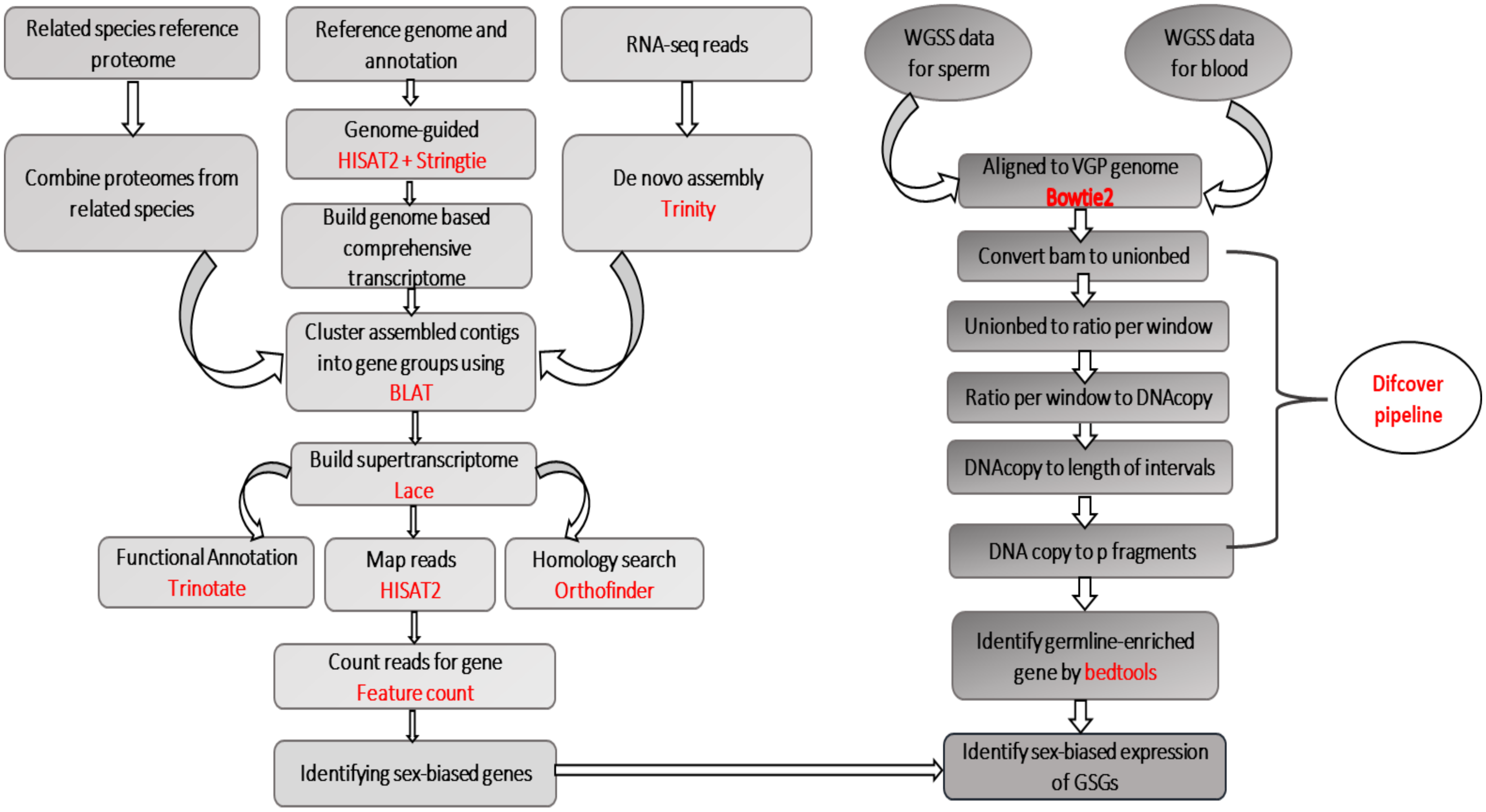

Supplementary Fig. 14: Step-by-step workflow and pipelines used for the study.

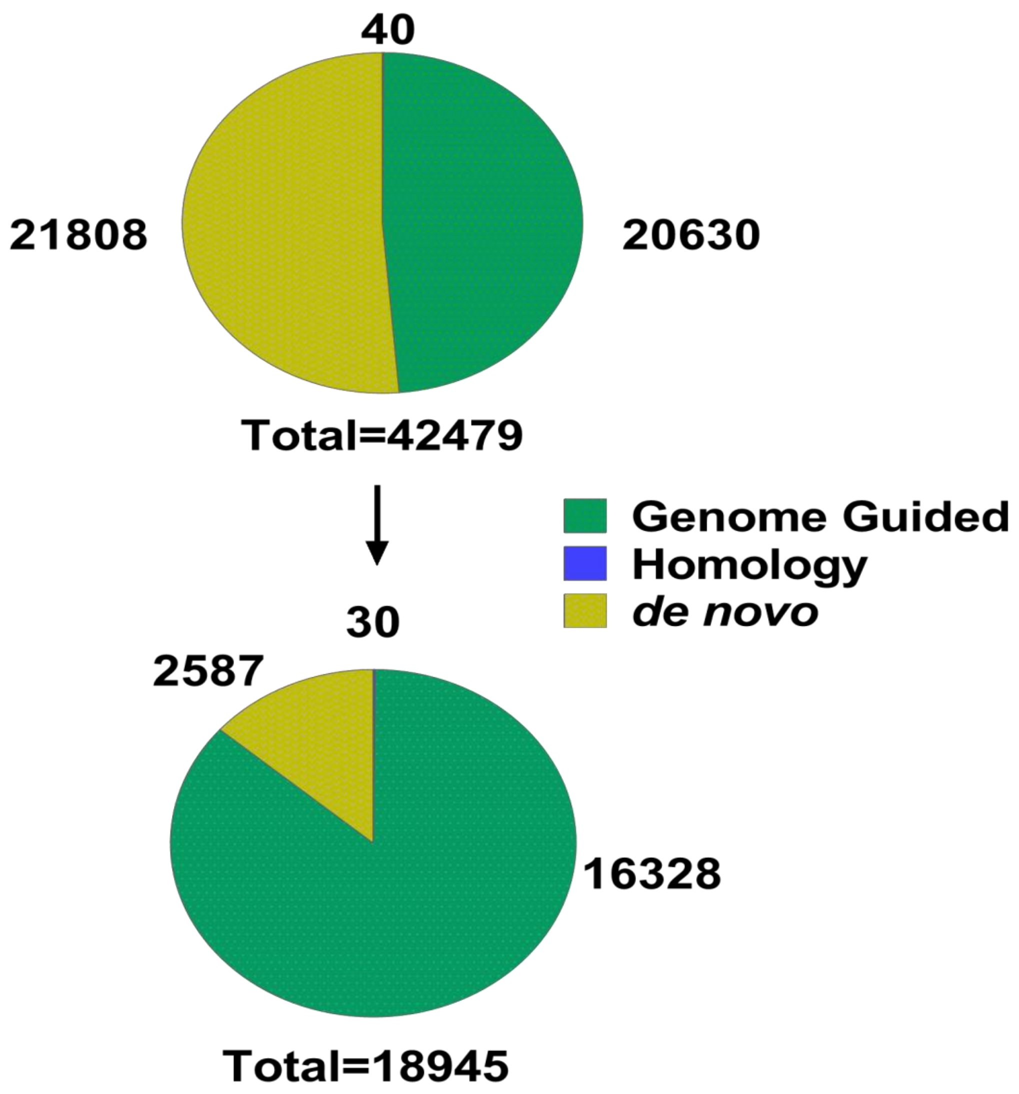

Supplementary Fig. 15: Pie charts showing the number of genes identified by the three-tiered necklace pipeline. Top pie chart shows the initial number of genes generated by the pipeline and the bottom chart shows the final number of post-filtered genes used for the study.

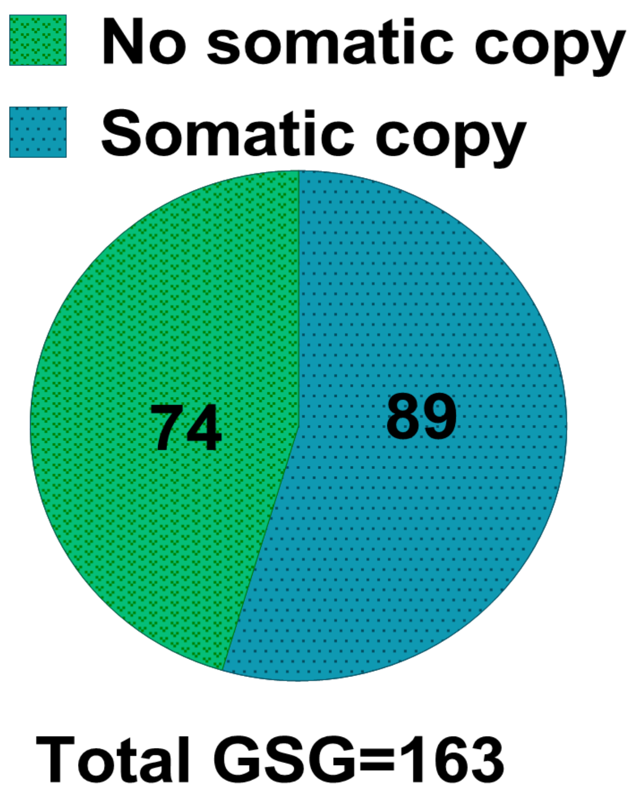

Supplementary Fig. 16: Pie charts showing the number of somatic and germline paralogues of GSGs

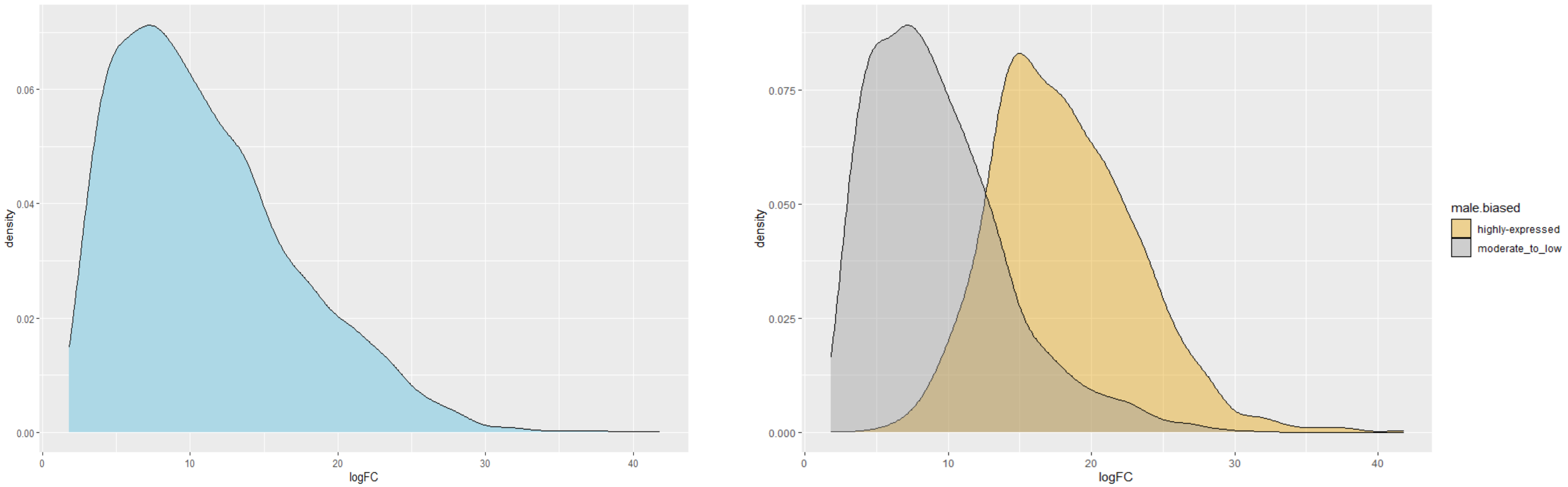

Supplementary Fig. 17: Density plots showing the overall logFC (log fold change) of a) all male-biased genes in the genome b) the density of genes classified as having high or moderate male-biased expression.

Supplementary Table 1: Details of sea lamprey (*Petromyzon marinus*) samples used in this study. Sex was determined by visual inspection of the gonad during dissection, stages of metamorphosis were identified according to the morphological criteria outlined in[1], and gonadal characteristics were inferred from sex, larval size, and life stage (see Supplementary Fig. 1[2]).

Sex	Stage	Sample ID	Sample Date	Length (mm)	Collection Site	Basin	Life Stage	Gonadal Characteristics
**Undetermined**	**Undifferentiated Larvae**	L_UD1	July 2016	57	Au Sable R, MI	Huron	Larval stage	Small, histologically undifferentiated gonad
		L_UD2	April 2018	66	Richibucto R, NB	Atlantic	Larval stage	" "
**Female**	**Early Female**	F_E1	April 2018	81	Chippewa R, MI	Huron	Larval stage	Ovarian differentiation in progress/completed; synchronous production of primary oocytes with the onset of meiosis I; majority of germ cells have become oocytes
		F_E2	April 2018	95	Chippewa R, MI	Huron	Larval stage	" "
	**Mid Female**	F_M1	Aug 2015	135	Richibucto R, NB	Atlantic	Metamorphosing stage 1	Oocytes arrested in meiotic prophase; cytoplasm grows at gradual rate
		F_M2	Aug 2015	130	Richibucto R, NB	Atlantic	Metamorphosing stage 1	" "
		F_M3	July 2016	128	Richibucto R, NB	Atlantic	Metamorphosing stage 3	" "
		F_M4	Aug 2017	120	Richibucto R, NB	Atlantic	Metamorphosing stage 4	" "
		F_M5	Nov 2015	129		Huron or Michigan[1]	Metamorphosing stage 7	" "
		F_M6	Oct 2017	130	Richibucto R, NB	Atlantic	Post-metamorphosis	" "
	**Late Female**	F_L1	June 2018	510	Ocqueoc R, MI	Huron	Late upstream migrant	Late sexual maturation; vitellogenesis complete, ovulation approaching/complete
		F_L2	June 2018	550	Ocqueoc R, MI	Huron	Late upstream migrant	" "
**Prospective Male**	**Prospective Male**	PM_1	April 2018	74	Richibucto R, NB	Atlantic	Larval stage	Small, histologically undifferentiated gonad beyond the size at which ovarian differentiation is in progress/complete
		PM_2	April 2018	75	Chippewa R, MI	Huron	Larval stage	" "
		PM_3	July 2018	82	Chippewa R, MI	Huron	Larval stage	" "
		PM_4	July 2018	99	Chippewa R, MI	Huron	Larval stage	" "
		PM_4	July 2017	118	Richibucto R, NB	Atlantic	Larval stage/ Metamorphosing stage 1	" "
**Male**	**Early Male**	M_E1	July 2017	132	Richibucto R, NB	Atlantic	Metamorphosing stage 1	Early stage of spermatogonial differentiation and production of Type A spermatogonia
		M_E2	Aug 2017	129	Richibucto R, NB	Atlantic	Metamorphosing stage 3	" "
		M_E3	Aug 2017	114	Richibucto R, NB	Atlantic	Metamorphosing stage 4	" "
		M_E4	Aug 2017	126	Richibucto R, NB	Atlantic	Metamorphosing stage 5	" "
		M_E5	Aug 2017	123	Richibucto R, NB	Atlantic	Metamorphosing stage 6	" "
	**Mid Male**	M_M1	Nov 2015	122		Huron or Michigan[1]	Metamorphosing stage 7	Undergoing spermatogonial proliferation and production of Type A and Type B spermatogonia
		M_M2	Nov 2015	133		Huron or Michigan[1]	Metamorphosing stage 7	" "
		M_M3	Dec 2017	130	Richibucto R, NB	Atlantic	Post-metamorphosis	" "
		M_M4	Dec 2016	114	Richibucto R, NB	Atlantic	Post-metamorphosis	" "
	**Late Male**	M_L1	April 2018	385	Black Mallard R, MI	Huron	Early upstream migrant	Early sexual maturation; spermatids, immature sperm
		M_L2	June 2018	400	Ocqueoc R, MI	Huron	Late upstream migrant	Late sexual maturation; mature sperm, spermiation approaching/complete

[1] Larval lampreys collected from multiple tributaries of Lake Huron and Lake Michigan and housed communally so specific sample site is not available.

Supplementary Table 2: List of sea lamprey GSGs and their tissue of bias that are associated with gonadal development, differentiation or sex determination in other taxa. NB denotes a lack of bias towards male or female.

Putative gene name	Bias: testes/ovary	Known function in gonad	Species	Reference
AGRL3	Testes	Female-biased expression	Tilapia	3
AR2BP	NB			
AVR2B	Testes			
CADH	Testes			
CADM2/3	Testes			
CFDP2	NB			
EPHA3	Testes			
HNRPL	Testes			
JAG2	NB			
LORF2	Testes			
LRRN1	NB			
NLRC3	NB			
PCD10	Testes			
PKHA5/6/7	NB			
RBM46	Testes			
SEM4D	Testes			
PPM1D	Testes	Male-biased expression	Tilapia	3
CCNJ	Testes			
WNT5A/5B	Testes			
BIRC1	Testes	Gonad development	Tilapia	4
CDC20	NB	Gonad development	Carp	4
AVR2A	Testes			
DDR48	NB			
RBP2A	Testes			
WDR78	Testes			
MYCN	Testes			
GTF2IRD2	Testes			
B4GALT1	Testes	Female-biased expression	Carp	3
DACH1	NB	Gonad development	Drosophila	
FAT1/3	Testes	Female-biased expression	Mouse	5
MSH4	Testes	Gonad development	Mouse	5
SYCP1	Testes			
FGF8B	Testes	Sex determination & differentiation	Mouse	6–8
RSPO3	Testes	Sex differentiation	Mammals	6–8
FGFR3	Testes	Sex determination and differentiation	Sturgeon	9
H90A1	NB	Sex-biased expression in male	Olive flounder	10
PTPRF	Testes	Gonad development	Medaka	11
PVRL1/4	NB			
RPAB4	NB	Gonad differentiation	Yellow carp	12

Supplementary References:

1. Manzon, R. G., Youson, J. H. & Holmes, J. A. Lamprey Metamorphosis. *Lampreys Biol. Conserv. Control* **1**, 139–214 (2015).
2. Docker, M. F., Beamish, F. W. H., Yasmin, T., Bryan, M. B. & Khan, A. The Lamprey Gonad. in *Lampreys: Biology, Conservation and Control* (2019). doi:10.1007/978-94-024-1684-8_1.
3. Tao, W. *et al.* Characterization of gonadal transcriptomes from Nile Tilapia (*Oreochromis niloticus*) reveals differentially expressed genes. *PLoS One* **8**, e63604 (2013).
4. Anitha, A. *et al.* Gonadal transcriptome analysis of the common carp, *Cyprinus carpio*: Identification of differentially expressed genes and SSRs. *Gen. Comp. Endocrinol.* **279**, 67–77 (2019).
5. Wang, J. *et al.* Comprehensive transcriptomic analysis of mouse gonadal development involving sexual differentiation, meiosis and gametogenesis. *Biol. Proced. Online* **21**, 20 (2019).
6. Makiyan, Z. Studies of gonadal sex differentiation. *Organogenesis* **12**, 42–51 (2016).
7. Windley, S. P. & Wilhelm, D. Signaling Pathways Involved in Mammalian Sex Determination and Gonad Development. *Sex. Dev.* **9,** 297–315 (2016).
8. Quinn, A. & Koopman, P. The molecular genetics of sex determination and sex reversal in mammals. *Semin. Reprod. Med.* **30**, 351–363 (2012).
9. Chen, Y. *et al.* Gonadal transcriptome sequencing of the critically endangered *Acipenser dabryanus* to discover candidate sex-related genes. *PeerJ* **2018**, e5389 (2018).
10. Fan, Z. *et al.* Gonadal transcriptome analysis of male and female olive flounder (*Paralichthys olivaceus*). *Biomed Res. Int.* **2014**, (2014).
11. Lai, K. P. *et al.* Transcriptomic analysis reveals transgenerational effect of hypoxia on the neural control of testicular functions. *Aquat. Toxicol.* **195**, 41–48 (2018).
12. Jia, Y. *et al.* Transcriptome analysis of three critical periods of ovarian development in Yellow River carp (*Cyprinus carpio*). *Theriogenology* **105**, 15–26 (2018).
