# Supplementary material for: Pervasive male-biased expression throughout the germline-specific regions of the sea lamprey genome supports key roles in sex differentiation and spermatogenesis
Source: Commun Biol. 2022 May 10;5:434. doi: 10.1038/s42003-022-03375-z (PMC9090840; doi:10.1038/s42003-022-03375-z)
Supplement: Supplementary file 3 — Description of Additional Supplementary Files [file 42003_2022_3375_MOESM3_ESM.pdf]

Description of Additional Supplementary Files

File name: Supplementary Data 1
Description: Depth of coverage ratios for all genomic intervals of germline-specific region. log2(standardized sperm coverage/blood coverage) greater than 2 is considered to be germline-specific region.

File name: Supplementary Data 2
Description: List of germline-specific genes with genomic location, enrichment score, putative gene name and normalized counts.

File name: Supplementary Data 3
Description: Number of somatic and germline paralogues of GSG in the genome and their expression.

File name: Supplementary Data 4
Description: PANTHER over representation test for molecular function.

File name: Supplementary Data 5
Description: REVIGO GO analysis for biological function.

File name: Supplementary Data 6
Description: List of somatic paralogoues of GSGs with their map location, chromosome number, their putative gene name as well as their normalized count.

File name: Supplementary Data 7
Description: Summary statistics of gene expression differences of somatic and GSR paralogoues of GSGs in different stages of males. EM denotes for Early male, MM for Mid male, LM for Late male am PM for Prospective male.

File name: Supplementary Data 8
Description: Proportion of male-biased genes along with their map location, putative gene name and enrichmentment score in different stages of males and females overall. Enrichment score would define which genes would fall under somatic genome and which gene would fall under germline genome.

File name: Supplementary Data 9
Description: Proportion of male to female in GSGs.

File name: Supplementary Data 10
Description: List of GSGs expressed in Pre- and Post-PGR embryo samples.

File name: Supplementary Data 11
Description: Orthogroup copy number of GSGs.
