# Supplementary material for: Pervasive male-biased expression throughout the germline-specific regions of the sea lamprey genome supports key roles in sex differentiation and spermatogenesis
Source: Commun Biol. 2022 May 10;5:434. doi: 10.1038/s42003-022-03375-z (PMC9090840; doi:10.1038/s42003-022-03375-z)
Supplement: Supplementary file 15 — Reporting Summary [file 42003_2022_3375_MOESM15_ESM.pdf]

nature portfolio

Corresponding author(s): *Double-blind peer review submissions: write DBPR and your manuscript number here instead of author names.*

Last updated by author(s): *YYYY-MM-DD*

Reporting Summary

Nature Portfolio wishes to improve the reproducibility of the work that we publish. This form provides structure for consistency and transparency in reporting. For further information on Nature Portfolio policies, see our Editorial Policies and the Editorial Policy Checklist.

Statistics

For all statistical analyses, confirm that the following items are present in the figure legend, table legend, main text, or Methods section.

n/a	Confirmed	
☐	☒	The exact sample size (*n*) for each experimental group/condition, given as a discrete number and unit of measurement
☐	☒	A statement on whether measurements were taken from distinct samples or whether the same sample was measured repeatedly
☐	☒	The statistical test(s) used AND whether they are one- or two-sided<br>*Only common tests should be described solely by name; describe more complex techniques in the Methods section.*
☐	☒	A description of all covariates tested
☐	☒	A description of any assumptions or corrections, such as tests of normality and adjustment for multiple comparisons
☒	☐	A full description of the statistical parameters including central tendency (e.g. means) or other basic estimates (e.g. regression coefficient) AND variation (e.g. standard deviation) or associated estimates of uncertainty (e.g. confidence intervals)
☐	☒	For null hypothesis testing, the test statistic (e.g. *F*, *t*, *r*) with confidence intervals, effect sizes, degrees of freedom and *P* value noted<br>*Give P values as exact values whenever suitable.*
☒	☐	For Bayesian analysis, information on the choice of priors and Markov chain Monte Carlo settings
☒	☐	For hierarchical and complex designs, identification of the appropriate level for tests and full reporting of outcomes
☒	☐	Estimates of effect sizes (e.g. Cohen's *d*, Pearson's *r*), indicating how they were calculated

Our web collection on statistics for biologists contains articles on many of the points above.

Software and code

Policy information about availability of computer code

Data collection	A variety of publicly available datasets were downloaded for analyses (please see research sample below), but no custom scripts were used for this.
Data analysis	FASTQC (v0.11.8), Trimmomatic (v0.36), Trinotate pipeline (v3.2.0), Transdecoder (v5.5.0), hmmer (v3.2.1),signalp (v4.1f), tmHMM (v2.0c), Rnammer (v1.2), DifCover pipeline (https://github.com/timnat/DifCover), HISAT2 (v 2.2.1), Stringtie (v 2.0), DESeq2 and EdgeR, PANTHER (v14) , REVIGO, Maaft

For manuscripts utilizing custom algorithms or software that are central to the research but not yet described in published literature, software must be made available to editors and reviewers. We strongly encourage code deposition in a community repository (e.g. GitHub). See the Nature Portfolio guidelines for submitting code & software for further information.

Data

Policy information about availability of data

All manuscripts must include a data availability statement. This statement should provide the following information, where applicable:

- Accession codes, unique identifiers, or web links for publicly available datasets
- A description of any restrictions on data availability
- For clinical datasets or third party data, please ensure that the statement adheres to our policy

The RNA-sequencing reads used for this study have been deposited in the NCBI repository under the BioProject accession number PRJNA749754 and will be available upon publication of manuscript.

Field-specific reporting

Please select the one below that is the best fit for your research. If you are not sure, read the appropriate sections before making your selection.

☐ Life sciences ☐ Behavioural & social sciences ☒ Ecological, evolutionary & environmental sciences

For a reference copy of the document with all sections, see nature.com/documents/nr-reporting-summary-flat.pdf

Ecological, evolutionary & environmental sciences study design

All studies must disclose on these points even when the disclosure is negative.

Study description

We analyzed RNA-sequence data from 28 sea lamprey gonads sampled across life-history stages, generated a genome-guided de novo superTransciptome with annotations, and identified genes in the germline-specific region. We have used RNA from individuals from different stages of gonadal development. They are early males (n = 4), mid males (n = 6), late males (n = 2), early females (n = 2), mid-stage females (n = 6), late females (n = 2), undifferentiated gonads (n = 2) and presumptive male larvae (n = 4).

Research sample

The novel research data is listed above. Additionally, we used publicly available genomics data: The sea lamprey reference germline genome and associated gene annotation file GCF_010993605.1_kPetMar1 available at NCBI, as well as sperm and blood DNA , Sperm SRR5535435, Blood SRR5535434. As well as embryo RNA-seq data: 1 dpf SRR3002837, 2 dpf (SRR3002840), 2.5 dpf (SRR3002843), 3 dpf (SRR3002846), 4 dpf (SRR3002849), 5 dpf (SRR3002852). For the orthofinder analyses, we employed proteomes from publicly available datasets at ENSEMBL: Spotted gar (Lepisosteus oculatus) GCF000242695.1_LepOcu1, Elephant shark (Callorhinchus milii) genome_assembly_id 49056), Human GRCh38, Mouse (Mus musculus) GRCm38, Zebrafish GRCz11, Chicken GRCg6a, Medaka (Oryzias sinensis) ASM858656v1, Spotted gar LepOcu1, Elephant shark Callorhinchus_milii-6.1.3, Coelacanths LatCha1, Hagfish (Eptatretus burgeri) Eburgeri_3.2, Amphioxus (Branchiostoma belcheri) GCF_001625305.1_Haploidv18h27 (available from NCBI).

Sampling strategy

The samples selected from the sea lamprey gonads were selected so as to have ~ 4 female and male samples per stage, for all possible stages. Some stages are very difficult to collect. These samples were provided to us by collaborators who collected the live lampreys for other studies for which ethics was approved. No live samples were handled by our research team.

Data collection

We received the gonad samples from three different collaborators (please see Supplementary Table 1 for full details) and then extracted RNA in our laboratory at the University of Manitoba. The extracted RNA was sent to Genome Quebec for RNA sequencing.

Timing and spatial scale

Sampling was performed based on the putative life history stage of the sea lamprey individuals based on their overall morphology and time of year of the sample. Full details are given in Supplementary Table 1.

Data exclusions

No data was excluded

Reproducibility

The data presented here were first analysed using a different reference, and the same primary observation was made: that a large number of genes exhibited reads only in male specimens and these reads were physically linked on scaffolds. The data and project were then transferred to use a chromosomal level assembly of the sea lamprey genome and the findings were reproduced.

Randomization

N/A

Blinding

Blinding was not relevant as the statistics used for the analyses were count and log fold changes that were generated by software. The sample ID's were also not informative so as to reduce bias.

Did the study involve field work? ☐ Yes ☒ No

Reporting for specific materials, systems and methods

We require information from authors about some types of materials, experimental systems and methods used in many studies. Here, indicate whether each material, system or method listed is relevant to your study. If you are not sure if a list item applies to your research, read the appropriate section before selecting a response.

Materials & experimental systems

n/a	Involved in the study	
☒	☐	Antibodies
☒	☐	Eukaryotic cell lines
☒	☐	Palaeontology and archaeology
☐	☒	Animals and other organisms
☒	☐	Human research participants
☒	☐	Clinical data
☒	☐	Dual use research of concern

Methods

n/a	Involved in the study	
☒	☐	ChIP-seq
☒	☐	Flow cytometry
☒	☐	MRI-based neuroimaging

Animals and other organisms

Policy information about studies involving animals; ARRIVE guidelines recommended for reporting animal research

Laboratory animals	*For laboratory animals, report species, strain, sex and age OR state that the study did not involve laboratory animals.*
Wild animals	The animals used in this study were collected from Au Sable River, Michigan; Richibucto River, New Brunswick; Chippewa River, Michigan; Black Mallard River, Michigan; or Ocqueoc River, Michigan. The samples were collected by collaborators J. Hume, N. Johnson or M. Wilkie, under ethics agreements they held with their respective institutional affiliations. The gonad tissue was then donated to us for our study.
Field-collected samples	*For laboratory work with field-collected samples, describe all relevant parameters such as housing, maintenance, temperature, photoperiod and end-of-experiment protocol OR state that the study did not involve samples collected from the field.*
Ethics oversight	An Abbreviated Protocol for Minimal Animal Involvement form completed at the University of Manitoba determined that an Animal Use Protocol (AUP) was not required because animals were not handled by us for the purposes of this project, nor were they sacrificed or manipulated solely to provide us with tissue.

Note that full information on the approval of the study protocol must also be provided in the manuscript.
